# Point-of-care lung ultrasound for the assessment of pneumonia: a narrative review in the COVID-19 era

**DOI:** 10.1007/s10396-020-01074-y

**Published:** 2021-01-13

**Authors:** Toru Kameda, Yoshihiro Mizuma, Hayato Taniguchi, Masato Fujita, Nobuyuki Taniguchi

**Affiliations:** 1 Department of Clinical Laboratory Medicine, Jichi Medical University, 3311-1 Yakushiji, Shimotsuke-shi, Tochigi, 329-0498 Japan; 2 Department of Emergency Medicine, Red Cross Society Azumino Hospital, 5685 Toyoshina, Azumino-shi, Nagano, 399-8293 Japan; 3 Department of Internal Medicine, Higashi Kobe Hospital, 1-24-13 Sumiyoshihonmachi, Higashinada-ku, Kobe-shi, Hyogo, 658-0051 Japan; 4 Advanced Critical Care and Emergency Center, Yokohama City University Medical Center, 4-57 Urafunecho, Minami-ku, Yokohama-shi, Kanagawa, 232-0044 Japan

**Keywords:** Lung ultrasound, Pneumonia, COVID-19

## Abstract

In the coronavirus disease-2019 (COVID-19) era, point-of-care lung ultrasound (LUS) has attracted increased attention. Prospective studies on LUS for the assessment of pneumonia in adult patients were extensively carried out for more than 10 years before this era. None of these prospective studies attempted to differentiate bacterial and viral pneumonia in adult patients using LUS. The majority of studies considered the LUS examination to be positive if sonographic consolidations or multiple B-lines were observed. Significant differences existed in the accuracy of these studies. Some studies revealed that LUS showed superior sensitivity to chest X-ray. These results indicate that point-of-care LUS has the potential to be an initial imaging modality for the diagnosis of pneumonia. The LUS diagnosis of ventilator-associated pneumonia in intensive care units is more challenging in comparison with the diagnosis of community-acquired pneumonia in emergency departments due to the limited access to the mechanically ventilated patients and the high prevalence of atelectasis. However, several studies have demonstrated that the combination of LUS findings with other clinical markers improved the diagnostic accuracy. In the COVID-19 era, many case reports and small observational studies on COVID-19 pneumonia have been published in a short period. Multiple B-lines were the most common and consistent finding in COVID-19 pneumonia. Serial LUS showed the deterioration of the disease. The knowledge and ideas on the application of LUS in the management of pneumonia that are expected to accumulate in the COVID-19 era may provide us with clues regarding more appropriate management.

## Introduction

Point-of-care ultrasound (POCUS), which is performed by clinicians at the bedside, has been developed and shared in many fields [1]. POCUS examinations focus on regions of interest based on patient history, vital signs and the results of physical examinations. The regions are assessed qualitatively or semi-quantitatively in time-sensitive situations [2–4]. POCUS can be used for wide range of purposes, including initial diagnoses, the assessment of severity, anatomic and physiological monitoring, and guidance for procedures [1].

The clinical application of lung ultrasound (LUS) by pulmonologists started in the latter half of 1970s, and the basis was established in 1980s in Japan [5]. This modality has mainly been used for the assessment of neoplasms and pleural effusion. In the last two decades, point-of-care LUS has been extensively studied and has attracted attention in other specialties, including emergency medicine [6–8], intensive care medicine [9–12], and hospital medicine [13]. LUS is now widely used in these fields for the assessment of pneumothorax and cardiogenic pulmonary edema and is supported by high-quality evidence [14, 15]. Meanwhile, the application of LUS in the assessment of pneumonia in adult and pediatric patients has been increasingly studied over the past decade [6, 16, 17]. In the coronavirus disease-2019 (COVID-19) era, this application has attracted increased attention [18–20].

Chest X-ray (CXR) is usually selected as the first imaging modality in patients with suspected pneumonia. However, several studies have reported that CXR shows poor sensitivity in detecting pneumonia [21]. Chest computed tomography (CT) has greater sensitivity than CXR in the diagnosis of pneumonia but is associated with increased cost and radiation exposure. Moreover, patients need to be transferred to the radiology department, potentially increasing the risk of disease transmission [22]. Recent studies indicate that LUS has the potential to reduce the use of CXR and chest CT for the assessment of pneumonia [6]. In this article, we comprehensively review the basis and value of LUS in the assessment of pneumonia in adult patients based on the clinical studies reported before and in the COVID-19 era.

## Image acquisition

LUS images can be acquired with a curvilinear probe, which is preferred in patients with obesity or a thick chest wall. A high-frequency linear probe is useful for detailed imaging of the pleural and subpleural pathologies.

LUS cannot provide a complete overview of the lung. The lungs are filled with air and surrounded by the bones of the thoracic cage. The air and bones interrupt the transmission of ultrasound beams. As a consequence, LUS is limited to the study of the pleura and subpleural lesions through the intercostal spaces. However, recent clinical studies have shown the high sensitivity of LUS in the diagnosis of pneumonia [6, 16, 17]. The fact indicates that inflammatory lesions or changes distribute peripherally or extend to the surface of the lung in the majority of pneumonia patients [23, 24]. LUS may show the part of the lesion or a clue to its existence in some patients [25, 26].

There is no best method or international standard for acquiring ultrasound images in cases of suspected pneumonia. If possible, the patient is examined in the sitting position. In general, the anterior, lateral, and posterior regions are comprehensively scanned [6, 16, 17]. Well-trained operators can perform a comprehensive LUS examination within 5 to 15 min [26–29]. In critically ill or immobile patients, it is not easy to fully scan the posterior region. In these cases, the probe is placed as posteriorly as possible [9], or the posterior region is scanned in the lateral decubitus position with assistance in order to improve the sensitivity of LUS [10, 28, 30]. In some cases, clinician operators may be able to limit the observed region under the guidance of auscultation or according to the location of pleural pain [2, 31]. When an abnormality is detected by LUS, the operator focuses on the area of interest to observe it in detail.

**Fig. 1:**
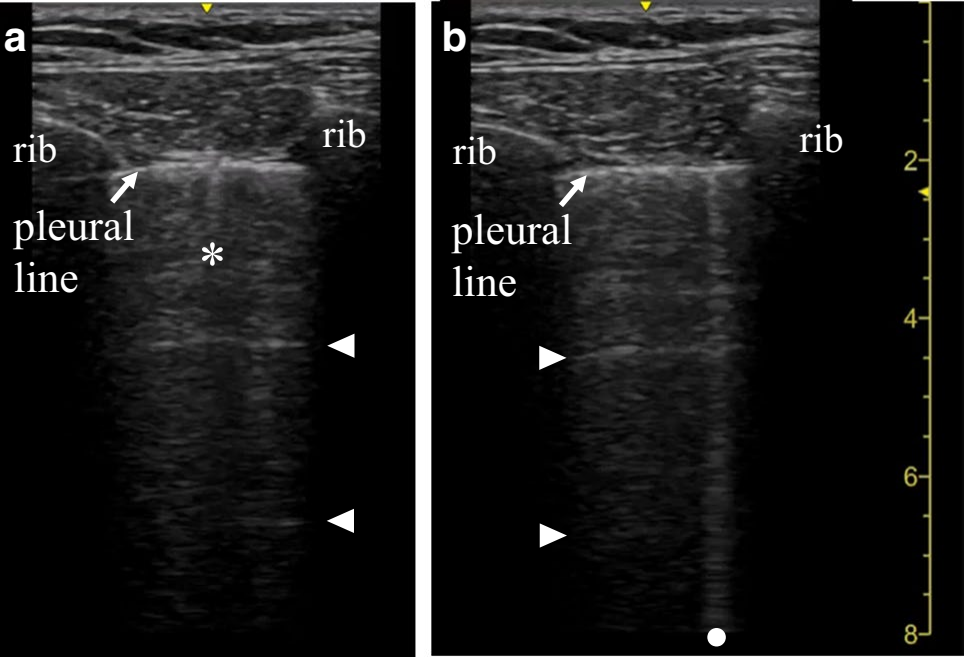
Longitudinal views of the anterior chest in a normal lung. The pattern created by the two ribs and pleural line is referred to as the ‘bat sign.’ A comet tail artifact (**a**, asterisk) and a B-line (**b**, dot) are shown just below the pleural line. Arrowheads indicate A-lines

The probe is put along the longitudinal lines on the chest to detect the interface between the parietal and visceral pleurae through the intercostal space (Fig. 1). The interface is detected as a thin horizontal hyperechoic line, which is called the ‘pleural line’ [9]. The thickness of the pleural line is affected by the reflection of the ultrasound beam by the subpleural air; thus, it does not anatomically correspond to the pleura. The pattern created by the two ribs and the pleural line is referred to as the ‘bat sign’ [9]. The bat sign is used to correctly identify the pleural line. Once the pleural line is identified, the probe is turned to be parallel to the ribs in the intercostal space to maximize the visualization of the pleural line. In normal lungs, ultrasound reveals the to-and-fro movement of the visceral pleura against the parietal pleura during respiration. The movement is called ‘lung sliding’ [9]. When an ultrasound beam is perpendicularly reflected on the pleural line, a reverberation artifact is created by repetitive reflection of the ultrasound beam between the pleural line and the footprint of the probe. Each line of this reverberation artifact is called an ‘A-line’ [9]. In the normal lung, vertical hyperechoic artifacts arising just below the visceral pleura are occasionally observed. The comet tail artifact is defined as a short artifact with attenuation. The ‘B-line’ artifact is defined as a laser-like artifact extending to the bottom of the screen without fading [2, 9]. Our previous study indicated that the physical basis of some B-lines is multiple reverberation [32].

Image quality is determined by machine settings such as presets and postimage processing. LUS usually relies on an analysis of artifacts, such as B-lines. The visualization of B-lines can be optimized by altering the machine settings [32, 33]. We should be aware that the configuration of B-lines is strongly affected by spatial compound imaging and the focal zone. Spatial compound imaging allows the acquisition of multiple coplanar images of the same object from different angles and combines them into a single image to enable the operator to obtain high contrast and high spatial resolution with reduced artifacts [34]. When interpreting B-lines, spatial compound imaging should be turned off to avoid counting B-lines erroneously [32]. The focal zone is the narrowest portion of the ultrasound beam and has the best lateral resolution. It is recommended that the focal zone be set at or near the level of the pleura in order to keep each B-line narrow for identification [32]. Some machines include presets specifically for LUS.

### Ultrasound findings of pneumonia

We herein describe the ultrasound findings of pneumonia that were established before the COVID-19 era.

### Multiple B-lines

**Fig. 2:**
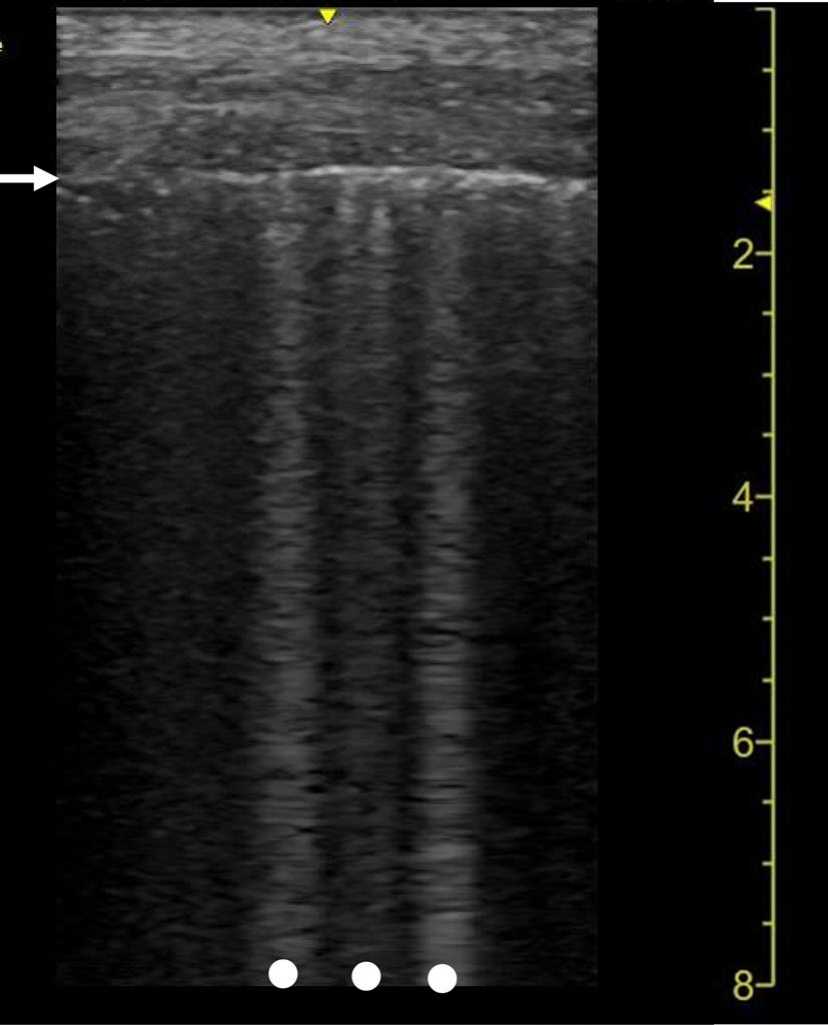
A lung ultrasound image of community-acquired pneumonia. Multiple B-lines (dots) are shown with irregularity of the pleural line (arrow)

When the air content decreases and the lung density increases in the lung surface, multiple B-lines become obvious and erase A-lines [2] (Fig. 2). As this change progresses, coalescent B-lines, which appear similar to white curtains, are observed. Several experimental and clinical studies indicate that B-lines originate from the accumulation of fluid just below the visceral pleura [35, 36], thickened interlobular septa [37], or deflation of the lung [38]. However, the sonographic–histologic correlation in B-lines has not been completely elucidated [32].

Multiple B-lines, including coalescent B-lines, are observed in many lung diseases [2]. The distribution of B-lines can aid in the differentiation of diseases. In general, multiple B-lines are diffusely distributed in patients with cardiogenic pulmonary edema, acute respiratory distress syndrome (ARDS), and interstitial lung diseases [2]. In patients with pneumonia, multiple B-lines are usually observed focally, multifocally, or patchily in ground glass opacities (GGOs) or around the areas of consolidation shown on CT images [27, 39, 40]. Multiple B-lines are not a specific finding of pneumonia; thus, this finding should be interpreted according to the clinical context.

### Sonographic consolidation

**Fig. 3:**
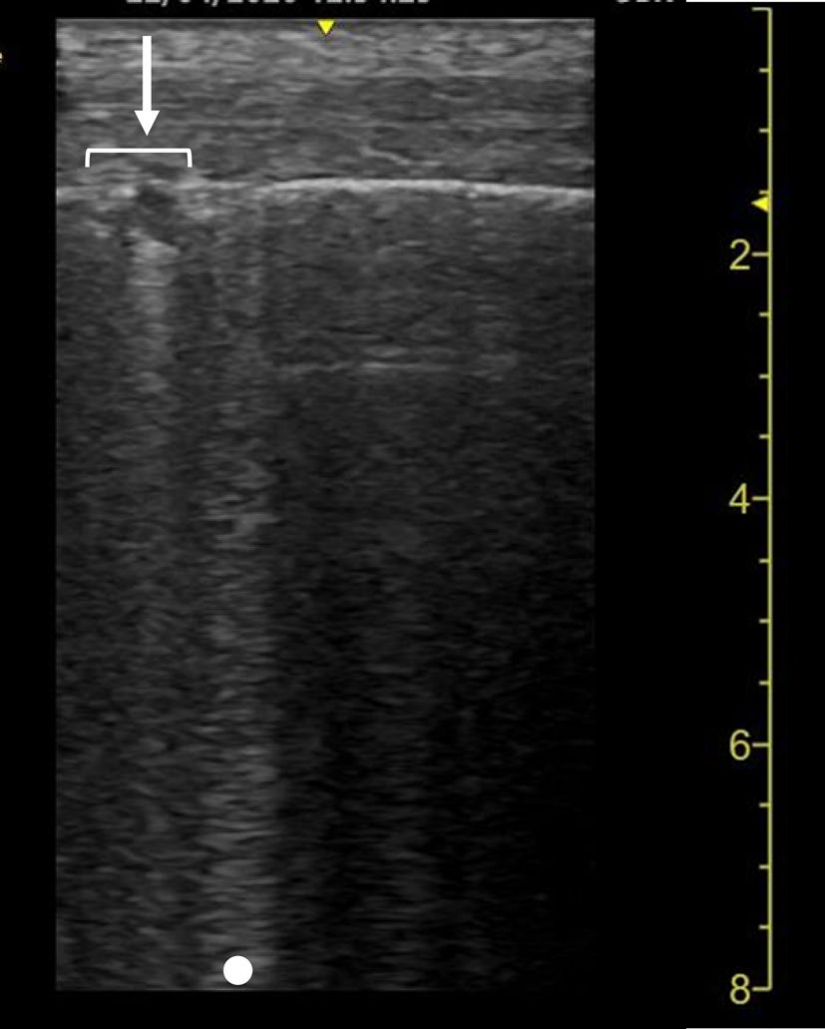
A lung ultrasound image of community-acquired pneumonia. A small subpleural consolidation (arrow) is shown with a B-line (dot). A normal sign is shown on the right side of the image

**Fig. 4:**
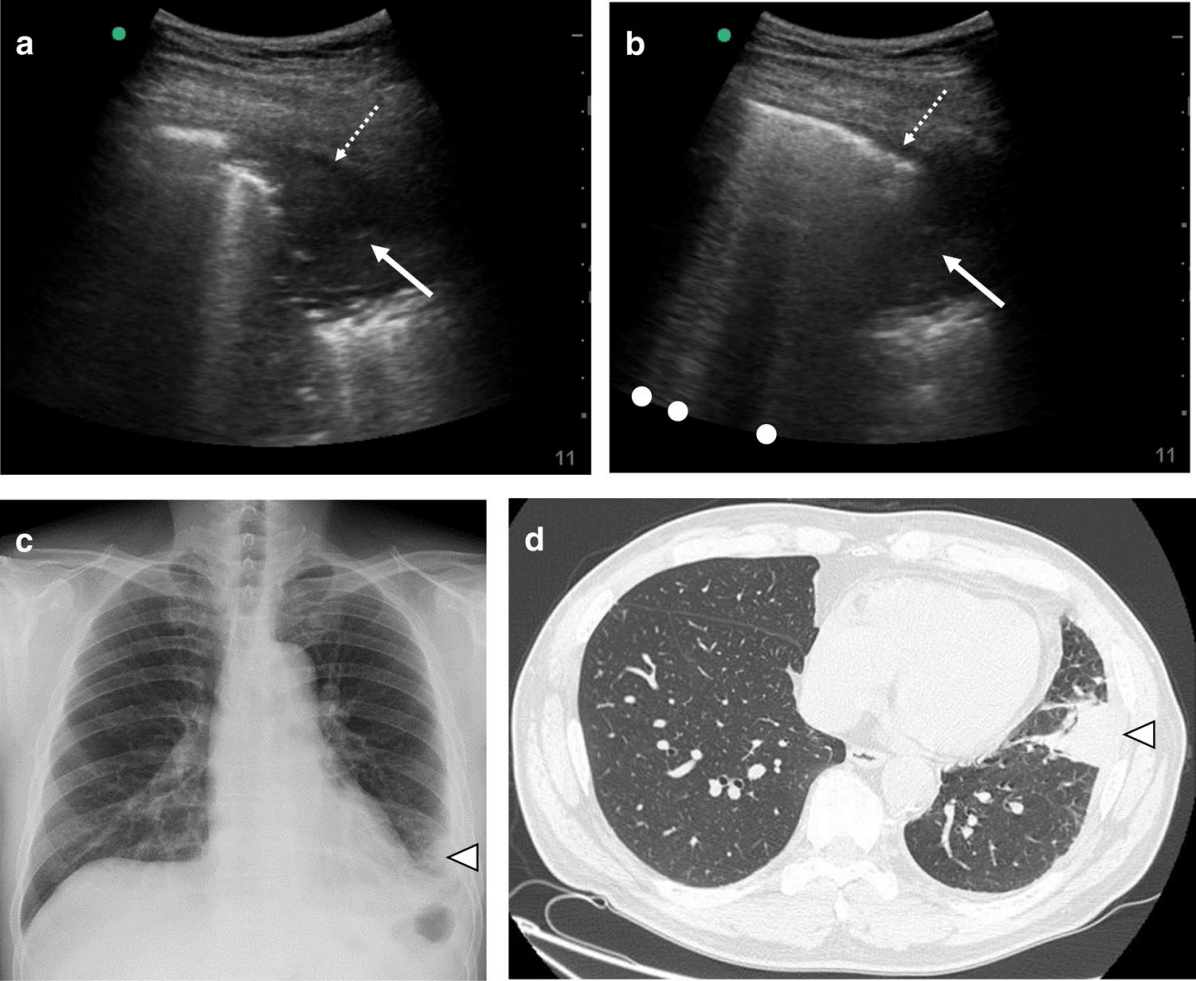
Lung ultrasound images (**a**, **b**), a chest X-ray image (**c**), and a chest computed tomography (CT) image (**d**) from a patient who presented with fever and left pleural pain. Lung ultrasound revealed sonographic consolidation (arrows) with slight pleural effusion (dotted arrows) in the left lower lateral chest, which corresponded to the site of the pleural pain. Multiple B-lines (dots) were observed around the site of sonographic consolidation. Chest X-ray and chest CT also revealed the consolidation (arrowheads). He was finally diagnosed with bacterial pneumonia

**Fig. 5:**
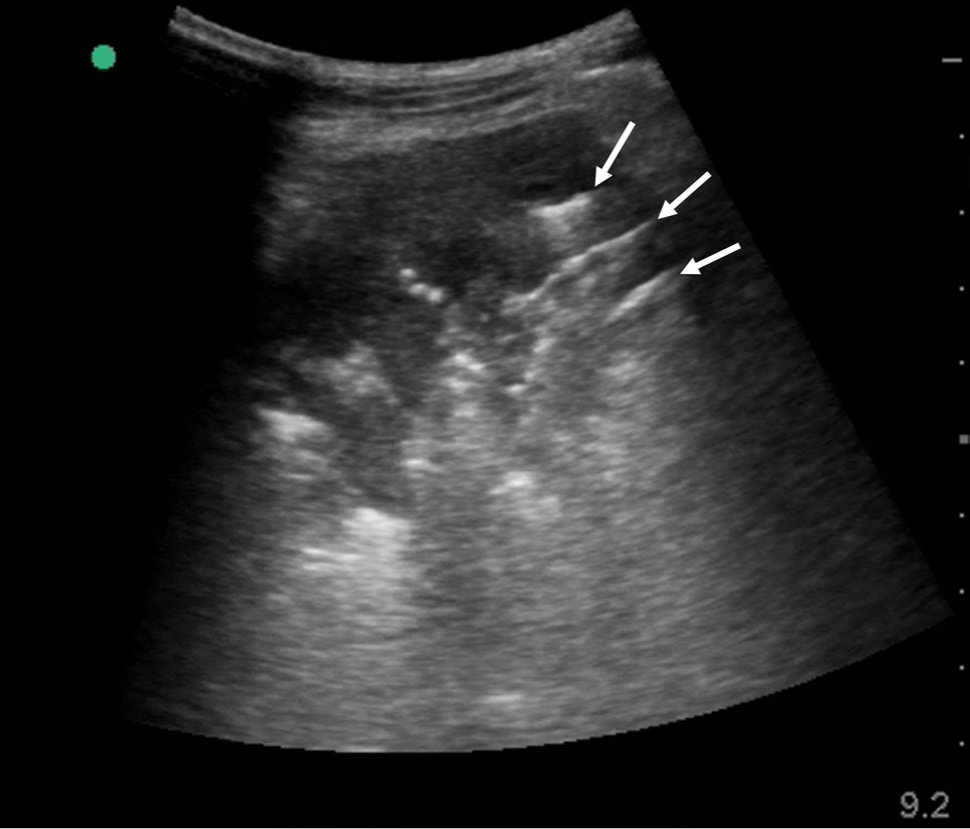
A lung ultrasound image of community-acquired pneumonia. A large lobar consolidation with irregular and serrated margins; the area of consolidation includes sonographic air bronchogram (arrows)

‘Sonographic consolidation’ is defined as a small subpleural hypoechoic region or large hypoechoic region with liver- or tissue-like echotexture (Figs. 3, 4, 5). Sonographic consolidations have various causes, including pneumonia, pulmonary embolism, lung carcinoma and metastasis, and atelectasis [2, 24, 41]. Sonographic consolidations in pneumonia are rarely rounded, as they would appear in patients with lung carcinoma or metastasis. The margins are usually irregular, serrated, and somewhat unclear [24]. In the presence of subpleural consolidation, the pleural line is not obvious, and lung sliding is reduced or absent [39].

The sonographic consolidations in pneumonia usually include high-echo spots and tree-like structures, which indicate air in the small bronchi. The tree-like structures seem to correspond to air bronchogram in radiology [24]. However, no study thus far has demonstrated the extent to which the tree-like structures on LUS correspond to air bronchogram on CT imaging [26]; thus, it is reasonable to use the term ‘sonographic air bronchogram’ at present in this context. Sonographic air bronchogram is not a specific sign of pneumonia [26]; however, movement with respiration is reported to be an important finding for differentiating pneumonia from obstructive atelectasis, in which the movement is not usually observed [42]. This phenomenon is called ‘dynamic air bronchogram’ [42].

LUS allows for follow-up care after the preliminary diagnosis of pneumonia and is capable of demonstrating whether the size of consolidations decreases after the initiation of treatment [25, 26]. When the dimensions of consolidations do not decrease or symptoms do not regress, follow-up LUS can help clinicians consider the possibility of malignancies or other diseases [26].

In addition to the B-mode image, color and power Doppler ultrasonography or contrast-enhanced ultrasonography is useful for evaluating the type of vascularization in consolidations for a differential diagnosis [5, 24]. However, with the exception of pulmonologists or radiologists, many clinicians are unfamiliar with these modalities in point-of-care LUS; thus, they are beyond the scope of this article.

### Pleural line abnormalities (irregularities)

The pleural line is recognized as a thin horizontal hyperechoic line in normal subjects. ‘Pleural line abnormality (irregularity)’ is defined as an irregularly thickened pleural line that can be observed in patients with pneumonia [23, 40], ARDS [2, 43], and interstitial lung diseases [44, 45]. Li et al. [44] reported on the application of patterns of pleural line abnormalities and their correspondence to CT findings for identifying specific pathologies. Pleural line abnormalities seem to be caused by several pathological changes. However, to the best of our knowledge, the sonographic–histologic correlation has not been investigated in pneumonia.

### Pleural effusion

Several studies have shown that pleural effusion is detected by LUS in 30–46% of pneumonia patients [26–28, 40]. In comparison with CT, LUS is an excellent modality for the detection of septations in pleural effusion [24].

### Clinical studies of LUS in the assessment of pneumonia before the COVID-19 era

Prospective studies on the application of LUS in the assessment of pneumonia (nonventilator associated) in adult patients were extensively carried out for more than 10 years before the COVID-19 era (Table 1) [9, 23, 25–31, 39, 40, 46–60]. These studies did not differentiate pneumonia based on the classification of pathogens or attempt to apply LUS in the differentiation of bacterial and viral pneumonia. The majority of the studies were performed in emergency departments (EDs). Other settings included intensive care units (ICUs), a stroke care unit, and an internal medicine ward. Many studies enrolled patients with suspected pneumonia/community-acquired pneumonia, acute dyspnea, or acute respiratory failure. In some studies that enrolled patients with acute dyspnea or acute respiratory failure, cardiac ultrasound and/or leg vein compression ultrasound was performed in addition to LUS to evaluate pneumonia and other pathologies causing these signs [9, 51, 53, 54, 57–59]. The majority of the studies considered an LUS examination to be positive if signs of sonographic consolidation and/or focal multiple B-lines were observed. In some studies, positivity was defined based on the presence of sonographic consolidation alone or B-lines alone. Some study groups distinguished between small subpleural consolidations (or lesions) and larger consolidations with sonographic air bronchogram (air bronchogram) [23, 31]. The evaluation of dynamic air bronchogram in an area of consolidation was adopted in some studies [23, 30, 31, 39, 40, 49]. The majority of studies used the final diagnosis, while some studies use chest CT [23, 29, 31, 47, 55, 56] or chest CT/CXR [25, 39, 48, 60] as a reference standard for the primary analysis.

**Table 1:** Characteristics of studies on lung ultrasound for the diagnosis of pneumonia

Study	Year	Setting	Inclusion criteria	N	Prevalence of pneumonia (%)	Sonographic findings used for the diagnosis
Lichtenstein et al. [9]	2008	ICU	ARF	260	32	Con, B-lines
Volpicelli et al. [46]	2008	ED	NS	217	14	B-lines
Parlamento et al. [39]	2009	ED	Suspected CAP	49	65	Con
Cortellaro et al. [27]	2012	ED	Suspected CAP	120	67	Con, B-lines
Reissig et al. [25]	2012	NS	Suspected CAP	362	63	NS
Nafae et al. [47]	2013	ICU	Suspected pneumonia	100	80	Con, B-lines
Unluer et al. [48]	2013	ED	Acute dyspnea	72	39	Con, B-lines
Bourcier et al. [49]	2014	ED	Suspected pneumonia	144	85	Con, B-lines
Daabis et al. [50]	2014	ICU	ARF	100	49	Con, B-lines
Laursen et al. [51]	2014	ED	Respiratory signs	158	35	NS
Busti et al. [28]	2014	SU	Suspected pneumonia	70	NS	Con, B-lines
Liu et al. [23]	2015	ED	Suspected CAP	179	62	Con, B-lines, PLA
Nazerian et al. [31]	2015	ED	Respiratory symptoms	285	30	Con
Pagano et al. [52]	2015	ED	Suspected pneumonia	105	65	Con, B-lines
Dexheimer et al. [53]	2015	ICU	ARF	37	46	Con, B-lines
Gallard et al. [54]	2015	ED	Acute dyspnea	130	21	Con, B-lines
Corradi et al. [55]	2015	ED	Suspected CAP	32	91	Con, B-lines
Taghizadieh et al. [56]	2015	ED	Suspected CAP	30	97	NS
Ticinesi et al. [30]	2016	ED	Suspected pneumonia	169	57	Con
Mantuani et al. [57]	2016	ED	Acute dyspnea	57	28	Con, B-lines
Zanobetti et al. [58]	2017	ED	Acute dyspnea	2683	40	Con, B-lines
Dimitrios et al. [59]	2017	ED	Acute dyspnea	115	4	B-lines
Interrigi et al. [60]	2017	ED	NS	370	19	Con
D’Amato et al. [26]	2017	Ward	X-ray-confirmed CAP	510	100	Con
Amatya et al. [29]	2018	ED	Suspected pneumonia	62	71	Con, B-lines
Sezgin et al. [40]	2020	ED	Suspected CAP	125	81	Con, B-lines

*ICU* intensive care unit, *ED* emergency department, *NS* not specified, *SU* stroke unit, *ARF* acute respiratory failure, *CAP* community-acquired pneumonia *N* number of included patients, *Con* consolidation, *PLA* pleural line abnormalities

Orso et al. 16 reported a systematic review and meta-analysis (SR/MA) that included 17 studies  [23, 25, 27, 31, 39, 46, 48, 49, 51, 52, 54–60], which evaluated the diagnostic accuracy of LUS for the diagnosis of pneumonia in ED patients of > 18 years of age. These studies provided a combined sample size of 5108 participants. The sensitivity ranged from 68% to 100%, and the specificity ranged from 25% to 100%. The pooled sensitivity and specificity were 92% (95% confidence interval [CI]: 86–95%) and 93% (95% CI 86–97%), respectively [16]. Other studies in ED settings were reported after the 17 studies. Amatya et al. [29] reported that the sensitivity and specificity were 91% and 61%, and Sezgin et al. [40] reported that the sensitivity and specificity were 98% and 96%, respectively. Several studies conducted in the ICU setting showed that the sensitivity ranged from 88% to 97%, and the specificity ranged from 50% to 94% [9, 47, 50, 53]. Some studies showed a small number of false-positive results in patients with neoplasia [26, 30, 31, 52], atelectasis [27, 31], pulmonary infarction [30, 52], abscess [27], congestive heart failure [52], chronic obstructive pulmonary disease [52], and fibrotic band [31]. As mentioned above, a significant difference in accuracy existed among these studies, and the misinterpretation of LUS findings may occur. Some studies compared the accuracy of LUS with CXR for the diagnosis of pneumonia (Table 2) [23, 27, 29–31, 40, 49, 52]. The sensitivity of LUS was better than that of CXR when using either the final diagnosis as a reference standard or the chest CT findings as a reference standard. These results indicate that point-of-care LUS has the potential to be an initial imaging modality for the diagnosis of pneumonia.

**Table 2:** The diagnostic accuracy of lung ultrasound and chest X-ray in the diagnosis of pneumonia

Reference standard	Final diagnosis				Chest CT			
	Sensitivity % (95% CI*)		Specificity % (95% CI*)		Sensitivity % (95% CI*)		Specificity % (95% CI*)	
	LUS	CXR	LUS	CXR	LUS	CXR	LUS	CXR
Cortellaro et al. [27]	99 (93–100)	67 (56–77)	95 (83–99)	85 (73–96)	96 (89–99)	69 (52–87)	NS	NS
Bourcier et al. [49]	95	60	57	76	100	52	NS	NS
Liu et al. [23]					95	78	99	94
Nazerian et al. [31]					81 (71–90)	64 (52–75)	94 (88–98)	90 (83–95)
Pagano et al. [52]	99 (94–100)	74 (67–80)	65 (56–67)	60 (47–71)	96 (91–99)	67 (67–74)	NS	NS
Ticinesi et al. [30]	92 (86–97)	47 (37–57)	94 (89–99)	93 (87–99)				
Amatya et al. [29]					91	73	61	50
Sezgin et al. [40]	98 (93–100)	88 (80–93)	96 (80–99)	92 (74–98)	98 (89–100)	90 (78–96)	92 (65–99)	83 (55–95)

*CT* computed tomography, *CI* confidence interval, *LUS* lung ultrasound, *CXR* chest X-ray, *NS* not specified

*CI is shown if it is described in the article

Two studies showed that the accuracy of LUS improved under the guidance of physical examinations. Reissig et al. [25] reported that a combination of auscultation and LUS findings increased the positive likelihood ratio (LR) and decreased the negative LR. Nazerian et al. [31] found that sensitivity and specificity increased in patients complaining of pleuritic chest pain, which allowed the initial examination to be focused on a limited chest area.

D’Amato et al. [26] showed the significance of LUS monitoring in pneumonia patients and reported the change in size of the areas of consolidation as follows: 6.3 ± 3.4 cm at 0 days, 2.5 ± 1.8 cm at 4–6 days, and 0.9 ± 1.4 cm at 9–14 days. Out of the 12 patients with delayed lesion healing, 7 were found to have lung cancer.

### Clinical studies for the assessment of ventilator-associated pneumonia

Ventilator-associated pneumonia (VAP) is a nosocomial infection in the ICU that affects patients who receive mechanical ventilation for more than 48 h. In mechanically ventilated patients, pulmonary infiltration on CXR frequently develops and may be associated with multiple etiologies, including VAP and noninfectious processes, such as atelectasis. A combination of clinical criteria, such as the Clinical Pulmonary Infection Score (CPIS), has been proposed for the diagnosis of VAP [61]. However, a previous study reported that CPIS showed poor diagnostic performance [62].

The LUS diagnosis of VAP in ICUs is more challenging in comparison with the diagnosis of community-acquired pneumonia in EDs because the mechanically ventilated patients cannot lie in the lateral decubitus position by themselves. The presence of extensive thoracic dressings or drainage tubes limits assessment by LUS in postoperative or trauma patients on mechanical ventilation. Furthermore, the higher prevalence of atelectasis in mechanically ventilated patients may cause lower specificity in the diagnosis of VAP [63]. However, LUS has a number of advantages in the ICU setting. It can be used immediately in real time at the bedside. In addition, it can also be used for sequential monitoring to detect the emergence of lesions [64] or to semi-quantify lung aeration [65]. Several studies have demonstrated the utility of LUS, based on the finding of sonographic consolidation, in the assessment of VAP in the ICU setting [63–69]. Some of the studies distinguished small subpleural consolidation (> 0.5 cm) and lobar/hemilobar consolidation with dynamic air bronchogram [63, 64, 68, 69].

Berlet et al. [67] reported that daily LUS examination with an abbreviated scanning protocol showed 100% sensitivity and 60% specificity in the diagnosis of VAP. The distinction between dynamic and static air bronchograms was of no significant additional benefit for the diagnosis in this study. They hypothesized that the lower specificity was explained by the high prevalence of atelectasis.

LUS alone cannot be used to accurately differentiate between inflammatory and noninflammatory consolidations. Zagli et al. [66] proposed a new score based on LUS, procalcitonin, culture of tracheal aspirate, purulence of tracheal secretion, temperature, and oxygenation to improve the diagnosis of VAP: the Chest Echography and Procalcitonin Pulmonary Infection Score (CEPPIS). A CEPPIS of > 5 was significantly better in predicting VAP (sensitivity, 81%; specificity, 85%) than LUS findings alone (sensitivity, 59%; specificity, 85%) or a CPIS of > 6 (sensitivity, 40%; specificity, 83%). Mongodi et al. [63] showed that the results were best with the combination of consolidations with dynamic air bronchogram or ≥ 2 areas with small subpleural consolidations and positive gram staining. Zhou et al. [68] demonstrated the superiority of the combination of LUS with procalcitonin to LUS alone in specificity. The sensitivity and specificity of LUS were 92% (95% CI 79–97%) and 63% (95% CI 51–74%), respectively, whereas the sensitivity and specificity of the combination were 81% (95% CI 67–91%) and 86% (95% CI 75–92%).

These studies excluded patients with ongoing pneumonia or other pulmonary diseases at ICU admission [63, 66–68]. Staub et al. [64] did not exclude such patients. As a result, there was a high frequency of sonographic consolidations before 48 h of mechanical ventilation. Instead, the emergence of consolidations was determined by comparison with the LUS findings of the previous day. They reported that serial LUS examinations detected the emergence of early and specific signs of VAP, and that the presence of sonographic consolidations was more specific indicators of VAP when they emerged in anterior lung areas, given that noninfectious infiltrates, such as passive or resorptive atelectasis, rarely compromise the anterior lung areas.

Bouhemad et al. [65] demonstrated that sonographic consolidation was replaced by B-lines when reaeration was confirmed by CT in patients treated with antibiotics, and LUS was more appropriate than CXR for quantifying lung reaeration.

A single-center diagnostic randomized controlled trial investigated whether LUS improved patient care. In the control group, VAP was diagnosed using a combination of CXR and clinical findings. In the intervention group, VAP was diagnosed using a combination of LUS as a daily monitoring tool and clinical findings. This trial showed that the index of ventilator-free days was higher in the intervention group than in the control group (7.8 ± 9.7 days versus 3.7 ± 6.4 days, p = 0.044). The use of LUS monitoring for the diagnosis of VAP may improve patient outcomes in comparison with the standard diagnostic strategy [69]. However, larger studies are needed to confirm the potential benefit of LUS for these patients.

### COVID-19 pneumonia

When considering the application of LUS in clinical practice, the CT features of patients with COVID-19 pneumonia provide us with useful information. An SR/MA of 13 studies on the anatomic distribution of the CT findings of patients with COVID-19 pneumonia found that the bilateral lungs (78%) and periphery (77%) were the most common sites of involvement. The incidence rates were higher in the right lower lobe (87%), left lower lobe (81%), and bilateral lower lobes (65%). The right upper lobe (65%), right middle lobe (55%), and left upper lobe (69%) were also commonly involved [70]. Based on these CT features, scanning of the entire region, including the posterior regions, is recommended when performing LUS for the evaluation of COVID-19 pneumonia. Lu et al. and Castelao et al. [71, 72] reported, based on an LUS study, that the lower lobes and posterior regions had a greater tendency to be involved.

**Fig. 6:**
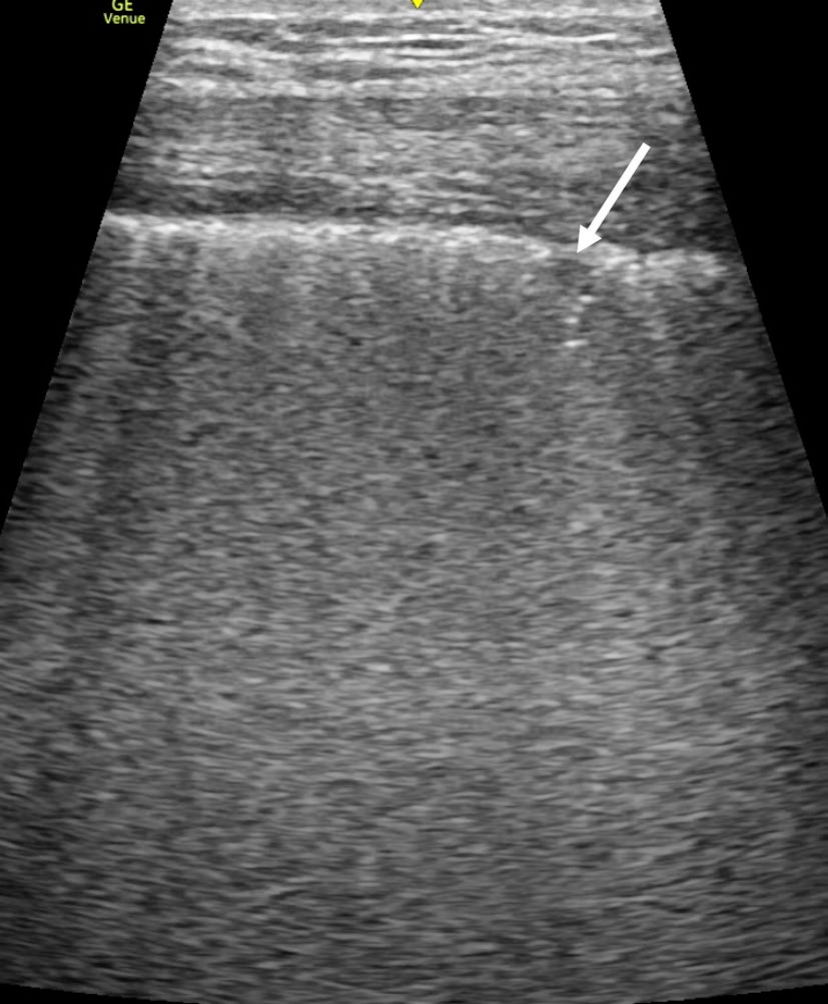
A lung ultrasound image of COVID-19 pneumonia showing coalescent B-lines with a small subpleural consolidation (arrow)

**Fig. 7:**
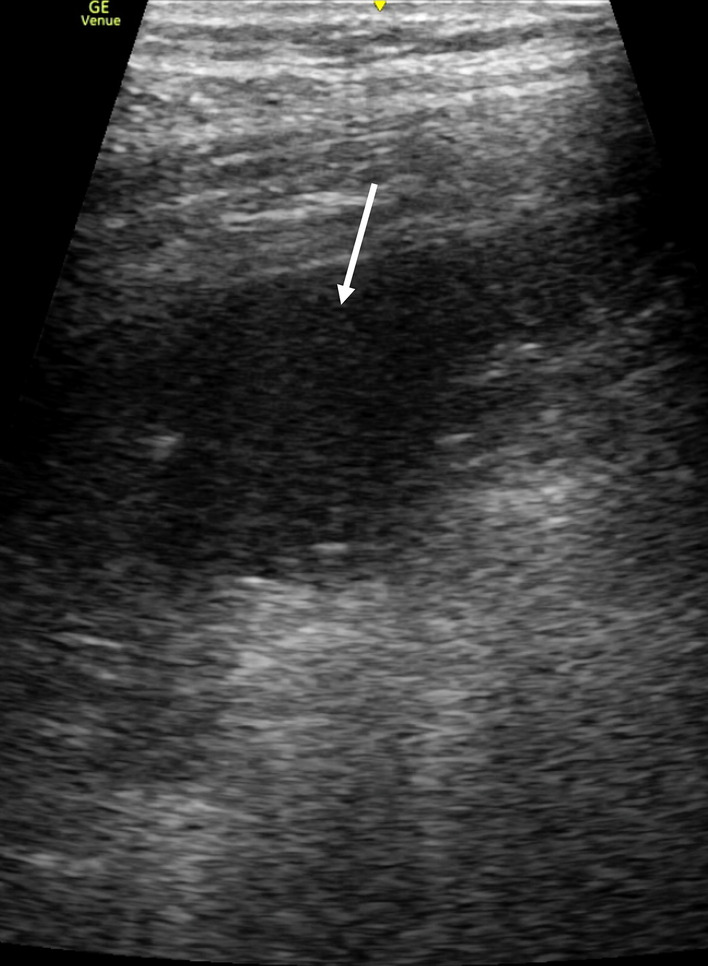
A lung ultrasound image of COVID-19 pneumonia showing sonographic consolidation in a gravitational region (arrow)

The LUS findings in COVID-19 pneumonia are similar to those described in patients with pneumonia before the COVID-19 era. Lopes et al. [73] showed an association between multiple B-lines on LUS and GGOs on CT as well as between subpleural consolidations on LUS and consolidations on CT. Mohamed et al. [74] reported an SR/MA including seven studies with a total of 122 COVID-19 patients which examined the role of LUS. The pooled proportion of multiple B-lines (including focal, multifocal, and coalescent types) (Fig. 6) detected by LUS was 0.97 (95% CI 0.94–1.00), that of pleural line abnormalities was 0.70 (95% CI 0.13–1.00), that of small subpleural consolidation (Fig. 6) or large consolidation (Fig. 7) was 0.39 (95% CI 0.21–0.58), and that of pleural effusion was 0.14 (95% CI 0.00–0.37). Multiple B-lines, including focal, multifocal, and coalescent types, were the most common and consistent findings; however, other LUS findings had a high degree of heterogeneity. Large lobar or trans-lobar consolidations with air bronchograms are less common in the early phases of COVID-19 pneumonia [20, 75]. When larger consolidations are observed initially, bacterial pneumonia or superimposed bacterial infection should be suspected [20, 75, 76]. Larger consolidations can be present in more advanced phases of COVID-19 pneumonia [75].

Interestingly, a newly described finding has been observed in patients in the early phases of COVID-19 pneumonia. Volpicelli et al. [20] named the finding the ‘light beam.’ This is a band-like vertical artifact that often appears and disappears from the screen with respiration. The artifact corresponds to the early appearance of GGO on CT. It is assumed that the light beam was included in the category of coalescent B-lines in some studies. A multicenter study is currently investigating the specificity of this sign [20].

A few studies have demonstrated high agreement of the severity between LUS and chest CT in severe patients with proven COVID-19 pneumonia [71, 77]. Yang et al. and Lopes et al. [73, 78] demonstrated that LUS was more sensitive than CT in the detection of subtle changes of the lung surface due to its high resolution; however, the clinical significance has not been proven. Meanwhile, a study demonstrated the diagnostic value of LUS using a COVID-19-positive result in a reverse transcriptase polymerase chain reaction (RT-PCR) assay as a reference standard. Peyrony et al. [79] reported the diagnostic accuracy of LUS with a pocket-sized device in 84 patients tested with RT-PCR in an ED. The sensitivity and specificity of a positive LUS result (defined as the presence of bilateral B-lines) were 77% (95% CI 62–88%) and 89% (95% CI 75–97%), respectively, and LUS was superior to CXR.

Time course of chest CT and LUS findings was reported in patients with COVID-19 pneumonia. Pan et al. [80] showed the time course of CT findings in 21 patients recovering from COVID-19 pneumonia without severe respiratory distress. Initial lung findings were small subpleural GGOs that grew larger with crazy-paving pattern (GGO with superimposed inter- and intralobular septal thickening) and consolidation. Lu et al. [71] reported that serial LUS mainly found B-lines to be increased and coalescent, and sonographic consolidations to be enlarged and increased, which was in high agreement with the CT findings. Extension toward larger consolidations, especially in a gravitational position (Fig. 7), indicates the phase of respiratory failure that requires invasive ventilation [81]. In the recovery phases, regression of the abovementioned findings can be observed with the re-emergence of A-lines [82, 83]. As mentioned above, serial LUS at the bedside is a promising method for monitoring COVID-19 pneumonia. Semi-quantification of lung aeration with LUS may be useful for monitoring the severity. Several semi-quantitative methods have been proposed [18, 20, 71, 84]. For example, each hemithorax is divided into six regions using anterior and posterior axillary lines and an axial line, and the following grades are used in each region: 0 points (A-lines and < 3 B-lines); 1 point (≥ 3 B-lines); 2 points (coalescent B-lines); 3 points (consolidation). The score is calculated as the sum of all regional scores ranging from 0 to 36 points [71].

Several studies indicate that baseline LUS score correlates with the eventual need for ICU admission and invasive mechanical ventilation, and the score is a predictor of mortality [85, 86]. The anterior region, although usually more spared, may have prognostic value because its involvement strongly correlated with the risk of requiring noninvasive respiratory support [72]. In deteriorating patients, LUS pathology worsened mostly in the anterior region [86].

Many case reports and observational studies with a small number of patients with COVID-19 pneumonia have been published in a short period. That is, the COVID-19 pandemic has made many clinicians interested in the usage of LUS for the assessment of COVID-19 pneumonia. However, to date, there is no robust evidence on the diagnostic accuracy, the efficiency as a monitoring tool, or the contribution to patients and medical personnel.

### Future perspectives of LUS in the evaluation of pneumonia in the COVID-19 era

Ultrasound had not been considered as a diagnostic tool for the evaluation of pneumonia. To date, the concept of POCUS has been widely accepted, and many clinical studies on LUS for the evaluation of pneumonia have been performed based on the idea of POCUS. Unlike chest CT, LUS cannot visualize the whole lung. However, several LUS findings obtained on or via the pleurae can contribute to the diagnosis of pneumonia and monitoring of the condition if they are properly interpreted in a clinical context.

Due to the superior sensitivity of LUS in comparison with CXR [23, 27, 29–31, 40, 49, 52, 65, 79], LUS has the potential to be an initial imaging modality or to reduce the number of CXR examinations [87]. It is also reasonable to consider the complimentary use of LUS, CXR, and chest CT owing to the excellent mobility of LUS at the bedside. LUS is a radiation-free imaging modality, which makes it useful for daily monitoring and particularly suitable for pregnant patients [88–90]. LUS with portable devices can also be performed in nursing homes [91] or patient homes [92] to reduce the need to transport the patient to a hospital for a diagnosis or monitoring with traditional imaging resources.

Theoretically, LUS may also be advantageous in infection control in the COVID-19 era. For example, stethoscope usage is strictly restricted to avoid COVID-19 virus transmission. It is difficult to create specific protective covers for stethoscopes or for medical personnel wearing personal protective equipment (PPE) to use a stethoscope [93]. The hazards of CT use include overuse of hospital resources, including PPE and other protective equipments that are required to safely perform CT, and disease transmission and exposure among staff engaged in patient transportation, technologists in imaging departments, and nonaffected patients who require CT examination in the same departments [22]. In contrast, portable ultrasound machines can easily be deployed at the bedside and covered with transparent plastic sheets for protection [94, 95]. Some handheld ultrasound devices with a wireless function are more easily covered with sheets or bags [93, 96]. However, it has to be kept in mind that the comprehensive LUS examination by experienced operators requires 5 to 15 min [26–29, 84]. It has not been scientifically evident whether introduction of LUS examination reduces the risk of the COVID-19 virus transmission.

When interest in LUS for the assessment of pneumonia is increasing, there are several issues to be considered and solved. First, some concerns have been raised regarding the robustness of the obtained results, because of the lack of a well-standardized methodology in the involved studies [16]. The issues in conducting clinical studies include the choice of reference standards for the final diagnosis, the competency of LUS examiners [84], and standardization of LUS findings. Therefore, more methodologically rigorous studies are still needed [16]. Second, there were few evidences on utility of LUS to differentiate bacterial pneumonia based on the classification of pathogens. To the best of our knowledge, there is only one case report [97] and few small clinical studies [98, 99] on LUS focused on viral pneumonia other than COVID-19 pneumonia in adult patients. Bacterial pneumonia is suspected when large lobar or trans-lobar consolidations are observed initially; however, there had been no prospective studies on the diagnostic accuracy of LUS in the differentiation of bacterial and viral pneumonia in adult patients before the COVID-19 era. On top of them, it has not been proven whether the features of LUS findings in COVID-19 pneumonia can be useful markers for differentiating COVID-19 pneumonia from bacterial pneumonia or viral pneumonia caused by other pathogens. Further studies are still needed to reveal whether LUS supports us to differentiate pneumonia based on the classification of pathogens. The robust evidence that is expected to accumulate in the COVID era may provide us with clues for the differentiation, which would facilitate more appropriate management. Third, standardization of the scanning regions and protocol has not been established in the assessment of pneumonia. Scanning all intercostal space is clinically time-consuming; therefore, the simplified approaches are needed, especially in time-sensitive situations. When selecting the scanning regions, it has to be kept in mind that distribution of the lesions is affected by pathogens and clinical course. On top of that, each approach suitable for the diagnosis, monitoring, or prediction of outcome may have to be developed according to the previous studies.

Utility of point-of-care LUS for the assessment of pneumonia has been shown mainly in the field of emergency medicine and intensive care medicine. Collaboration between these specialties and pulmonology is indispensable for the further development of this modality.

## Conclusions

LUS has received increased attention in the COVID-19 era. Before this era, many prospective studies were conducted on the use of LUS in the assessment of pneumonia. Significant differences existed in the accuracy of these studies. Some studies revealed that LUS showed superior sensitivity to chest X-ray. These results indicate that point-of care LUS has the potential to be an initial imaging modality for the diagnosis of pneumonia. In the COVID-19 era, many case reports and small observational studies on COVID-19 pneumonia have been published in a short period. However, to date, there is no robust evidence on the diagnostic accuracy of LUS, its efficiency as a monitoring tool, or its contribution to patients with COVID-19 pneumonia or the medical personnel engaged in their care. The knowledge and ideas related to the application of LUS in the management of pneumonia that are expected to accumulate in the COVID-19 era may provide us with clues that can facilitate more appropriate management.
